# Serum starvation-induced cell cycle synchronization stimulated mouse rDNA transcription reactivation during somatic cell reprogramming into iPSCs

**DOI:** 10.1186/s13287-016-0369-1

**Published:** 2016-08-11

**Authors:** Qiaoshi Zhao, Yanshuang Wu, Zhiyan Shan, Guangyu Bai, Zhendong Wang, Jing Hu, Li Liu, Tong Li, Jingling Shen, Lei Lei

**Affiliations:** Department of Histology and Embryology, Harbin Medical University, Xuefu Road 194#, Nangang District, Harbin, 150081 China

**Keywords:** Serum starvation, rDNA, Transcription activation, Reprogramming, Mouse embryonic fibroblast, Induced pluripotent stem cell

## Abstract

**Background:**

rDNA, the genes encoding ribosomal RNA (rRNA), is highly demanded for ribosome production and protein synthesis in growing cells such as pluripotent stem cells. rDNA transcription activity varies between cell types, metabolism conditions, and specific environmental challenges. Embryonic stem cells (ESCs), partially reprogrammed cells, and somatic cells reveal different epigenetic signatures, including rDNA epigenetic marks. rDNA epigenetic characteristic resetting is not quite clear during induced pluripotent stem cell (iPSC) generation. Little is known that whether the different rDNA epigenetic status in donor cells will result in different rDNA transcription activities, and furthermore affect reprogramming efficiency.

**Methods:**

We utilized serum starvation-synchronized mouse embryonic fibroblasts (MEFs) to generate S-iPSCs. Both MEFs and serum-refeeding MEFs (S-MEFs) were reprogrammed to a pluripotent state. rDNA-related genes, UBF proteins, and rDNA methylation levels were detected during the MEF and S-MEF cell reprogramming process.

**Results:**

We demonstrated that, after transient inhibition, retroviral induced rRNA transcriptional activity was reprogrammed towards a pluripotent state. Serum starvation would stimulate rDNA transcription reactivation during somatic cell reprogramming. Serum starvation improved the methylation status of donor cells at rRNA gene promoter regions.

**Conclusions:**

Our results provide insight into regulation of rDNA transcriptional activity during somatic cell reprogramming and allow for comparison of rDNA regulation patterns between iPSCs and S-iPSCs. Eventually, regulation of rDNA transcriptional activity will benefit partially reprogrammed cells to overcome the epigenetic barrier to pluripotency.

**Electronic supplementary material:**

The online version of this article (doi:10.1186/s13287-016-0369-1) contains supplementary material, which is available to authorized users.

## Background

rDNA, the genes encoding ribosomal RNA (rRNA), is highly demanded for ribosome production and protein synthesis in growing cells. Because the ribosome supply for protein synthesis is tightly linked to cell growth and proliferation, highly proliferating cells such as tumor and pluripotent stem cells adjust to active rRNA gene transcription. The rRNA gene encodes a precursor rRNA (45S pre-rRNA) that can be processed and posttranscriptionally modified to generate the mature 18S, 5.8S, and 28S rRNA [1, 2]. Transcription of rDNA by RNA polymerase I (Pol I) begins with the formation of a preinitiation complex on the promoter; that is, the interaction of upstream binding factor (UBF) and promoter selectivity factor—SL1 represents *Homo sapiens* and TIF-IB represents *Mus musculus* [3–7]. UBF activates rRNA gene transcription by recruiting Pol I and SL1/TIF-IB to the rDNA promoter [8]. As a basal regulatory factor, TIF-IA is cooperated with SL1/TIF-IB and drives the assembly of productive transcription initiation complexes [9, 10].

rDNA exists in three independent epigenetic states: active rDNA promoters are hypomethylated and marked by euchromatic histone modifications, silent rDNA promoters are hypermethylated and accompanied by heterochromatic features, and poised rDNA promoters display bivalent chromatin modifications which are permitted to reactivate [11, 12]. Nearly any unfavorable circumstance that slows cell growth or proliferation, such as nutrient or growth factor starvation, senescence, and toxic lesion, leads to a decrease in rDNA transcription and protein synthesis. Conversely, rDNA transcription is upregulated upon reversal of such conditions and by agents that stimulate growth [13, 14].

Dramatic epigenetic landscape remodeling is predestined in the process of somatic cell reprogramming to pluripotency [15, 16]. The stabilized self-sustained pluripotent state is believed to require several critical epigenetic modifications, including the rDNA specific regulatory mechanisms [17]. Recent research claimed that pluripotency factor OCT4 interacted with rDNA in both mouse and human embryonic stem cells (ESCs). An additional 17 pluripotency-associated transcription factors and three Polycomb proteins associated with rDNA in mouse ESCs, such as SOX2, NANOG, KLF4, STAT3, SMAD1, and C-MYC, suggested that pluripotency factors may regulate rRNA expression [18]. Zheng et al. [19] found that rRNA genes were not fully activated upon nuclear transfer, a nuclear reprogramming strategy. Practically, embryonic stem cell nuclear transfer (ESNT), cumulus cell nuclear transfer (CCNT), and mouse embryonic fibroblast nuclear transfer (MEFNT) embryos had different rDNA activities. The different rDNA activities of ESNT, CCNT, and MEFNT embryos were determined by the rDNA epigenetic status of donor cells.

Comparison of genome-wide epigenetic signatures between ESCs, partially reprogrammed cells, and diversified somatic cell types reveals differences between pluripotent and differentiated states. However, rDNA epigenetic characteristic resetting is not quite clear during induced pluripotent stem cell generation. We also want to know whether the different rDNA epigenetic status in donor cells will result in different rDNA transcription activities in retrovirus-induced reprogramming, and furthermore affect reprogramming efficiency. Here, we utilized serum starvation pretreated mouse embryonic fibroblasts (MEFs) to generate induced pluripotent stem cells (S-iPSCs). We demonstrated that serum starvation would stimulate rDNA transcription reactivation during somatic cell reprogramming. Our results provide insight into regulation of rDNA transcriptional activity during somatic cell reprogramming and allow for comparison of rDNA regulation patterns between induced pluripotent stem cells (iPSCs) and S-iPSCs. Eventually, regulation of rDNA transcriptional activity will benefit partially reprogrammed cells to overcome the epigenetic barrier to pluripotency.

## Methods

### Animals and cell culture

All mice used were bought at 6–8 weeks of age from Vital River (Beijing, China). Animal handling was in accordance with the Guidelines for the Care and Use of Laboratory Animals. Experiments were performed under the code of Practice Harbin Medicine University Ethics Committees.

B6D2F1 MEFs were prepared from E13.5 embryos of a C57BL/6 × DBA/2 background. MEFs used for iPSC derivation were cultured in DMEM plus 10 % FBS (Gibco). Partial MEFs, with the same passage as already described, were kept in DMEM plus 0.5 % FBS for 18 h as serum starvation treatment. After serum deprivation, MEFs were counted and passaged. For every 35 cm dish, 1 × 10^5^ MEFs and serum-starved MEFs were planted and refed with fresh serum (15 % FBS) for 16 h before retroviral infection. The normal MEFs and serum-refeeding MEFs (S-MEFs) were used as donor cells for iPSC generation. MEFs used in this study were maintained within three passages.

The mouse R1 ES cell line was purchased from American Type Culture Collection (http://www.atcc.org/). R1 ESCs and iPSCs were cultured on a feeder layer of mitotically inactivated MEFs with DMEM (Gibco) containing 15 % FBS (Gibco), 1000 U/ml LIF (Millipore), 1 mM sodium pyruvate (Sigma), 2 mM glutamine (Sigma), 0.1 mM 2-mercaptoethanol (Sigma), and 0.1 mM nonessential amino acids. For iPSC derivation, knockout DMEM (Gibco) and 20 % knockout Serum Replacement (KOSR; Gibco) were used instead of DMEM with 15 % FBS. R1 ESCs and iPSCs were passaged every 2 days.

### Generation of iPSCs and S-iPSCs

Retroviral production and infection were constructed as described previously [20]. In brief, the four retroviral vectors (pMXs-Oct4, Sox2, Klf4, and c-Myc) were introduced into plat-E cells using lipofectamine 2000 transfection reagent (Invitrogen). After 24 and 48 h, the virus-containing supernatants were harvested and concentrated. For standard infection in a 35-mm dish, 1 × 10^5^ donor cells were incubated with four retroviruses supplemented with 4 mg/ml polybrene for 24 h. The virus-containing medium was then replaced with the second supernatant for another 24 h (Day 0). Two days after infection (Day 2), the infected fibroblasts were replated with 2.5 × 10^4^ cells on mitomycin-C-treated MEF feeder layers and cultured in iPSC derivation medium. After the first mature colonies appeared, cell clumps were transferred onto feeder cells. After one or two passages, iPSCs or S-iPSCs were replated onto the feeders and ESC medium was changed every 24 h.

### Immunofluorescence analysis

Cells were fixed with 4 % paraformaldehyde for 30 min and then permeabilized with 0.5 % Triton X-100 for 15 min followed by blocking with 1 % BSA (Sigma). Cells were incubated in primary antibody overnight at 4 °C and in secondary antibody at room temperature for 1 h. The following primary antibodies were used: anti-Oct4, anti-Nanog, and anti-SSEA-1. The nuclei were stained with 1 μg/ml 4′,6-diamidino-2-phenylindole (DAPI; Sigma) for 30 s.

### Alkaline phosphatase staining and staining-positive colony counting

Alkaline phosphatase (AP) staining was performed with the BCIP/NBT Alkaline Phosphatase Colour Development Kit (Beyotime) according to the manufacturer’s instructions. Three independent dishes (35 mm) were used for counting AP staining-positive (AP^+^) colonies. For every dish, 10 visual fields by light microscopy were picked up randomly. The average of AP^+^ colonies was analyzed by the Image J analyzer system (http://imagej.nih.gov/ij/).

### Teratoma formation and histological analysis

Passage 8 (P 8) S-iPSCs (1 × 10^6^) were injected into the subcutaneous flanks of the nude mice. Four weeks later, the mice were euthanized and the tumors were fixed and sliced. The differentiation potential of S-iPSCs was confirmed by hematoxylin and eosin (H & E) staining.

### Reverse transcription and quantitative PCR analysis

To detect rDNA-related gene expression during the MEF reprogramming process, RNA was isolated from two types of MEFs during the iPSC generation procedure (Days 0, 3, 6, 9, and 12). Total RNA was isolated using TRIzol reagent (Invitrogen) and first-strand complementary DNA was synthesized using a High Capacity cDNA Reverse Transcription kit (ABI) according to the manufacturer’s instructions. Quantitative PCR (Q-PCR) was performed using SYBR green (Transgene) on a CFX96 Realtime System (Bio-Rad). Reactions were achieved in triplicate. Ct values were calculated using the 2^–ΔΔCt^ method and the expression of target genes were normalized to Gapdh expression. Primer sequences for each gene are listed in Additional file 1: Table S1.

### Western blotting

To test UBF protein expression during the somatic cell reprogramming process, MEFs (Days 0, 3, 6, 9, and 12), S-MEFs (Days 0, 3, 6, 9, and 12), iPSCs-A1, S-iPSCs-B3, and R1 ESCs were collected. Whole cell extracts were separated on 8 % SDS-polyacrylamide gel and transferred to nitrocellulose membranes. Membranes were then blocked in PBS-Tween (PBS-T) containing 5 % milk for 30 min at room temperature and then incubated with primary antibody solution at 4 °C overnight. After washing with PBS-T, the membrane was incubated with appropriate secondary antibodies. The protein levels of UBF and GAPDH were determined using their respective specific antibodies and visualized using the ECL kit according to the manufacturer’s instructions. Blots were quantified using the image J analyzer system (http://imagej.nih.gov/ij/).

### rDNA methylation analysis

To investigate rDNA methylation levels of donor cells, partially reprogrammed cells and iPSCs, MEFs, S-MEFs, Day 6 MEFs, Day 6 S-MEFs, iPSCs-A1, S-iPSCs-B3, and R1 ESCs were collected. Genomic DNA was converted using the EZ DNA Methylation-Direct™ kit (ZYMO Research, Irvine, CA, USA) according to the manufacturer’s instructions. rDNA promoter was PCR amplified using primers TAGTTTATTTTTTTTATTGGTTTGG (forward) and TAACATAAACACTTAAACACCACAA (reverse) as designed previously by our laboratory [19]. The PCR products were cloned into T3 vectors. At least 10 randomly selected clones for each sample were sequenced and analyzed.

### Statistical analysis

All experiments were carried out in triplicate and data are presented as mean ± SD for statistical comparison. A two-group comparison was performed using the Student’s *t* test, and significance of differences was assessed by *p* < 0.05.

## Results

### Generation of iPSCs from S-MEFs

**Fig. 1:**
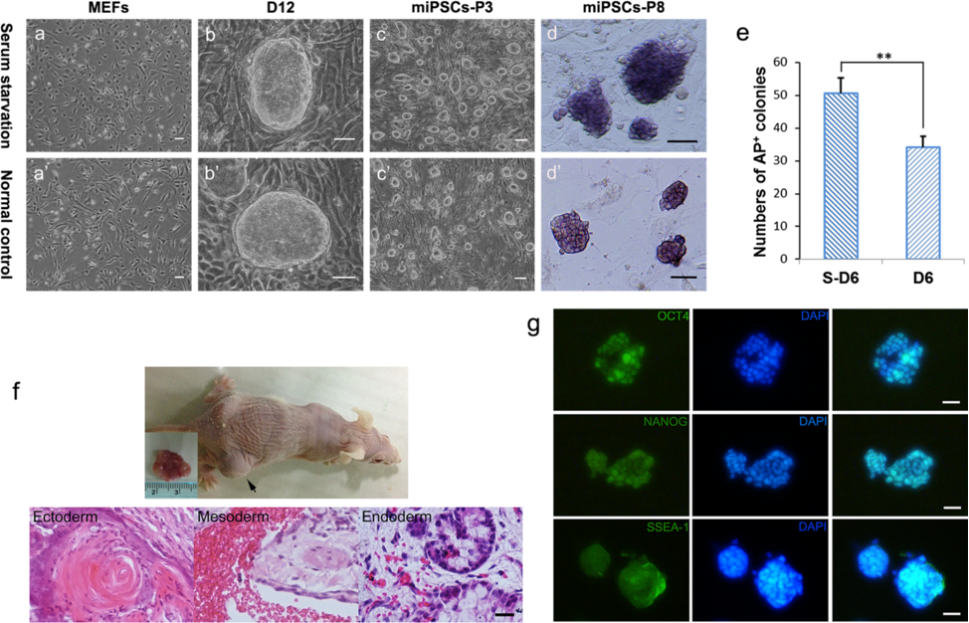
Morphological comparison of iPSCs derived from two types of MEFs and the identification of pluripotency. **a**, **a′** Morphology of S-MEFs and MEFs. **b**, **b′** Primary iPSC colony formed 12 days after transduction. **c**, **c′** Established P 3 iPSC clones. **d**, **d′** AP staining performed for P 8 iPSCs. **e** Statistical analysis of AP^+^ colonies generated from Day 6 pre-iPSCs during the reprogramming process. **Significant differences at *p* < 0.01. **f** Identity differentiation of mouse S-iPSCs in vivo. The nude mouse was injected with S-iPSCs (*black arrow* indicates the teratoma). Teratoma with all three germ layers determined by H & E staining was observed. **g** Immunofluorescence of pluripotency marker genes (Oct4, Nanog, and SSEA-1) in S-iPSCs. Nuclei were counterstained with DAPI. Scale bars = 25 μm. *AP* alkaline phosphatase, *D* day, *iPSC* induced pluripotent stem cell, *MEF* mouse embryonic fibroblast, *P* passage

In this study, B6D2F1 MEFs were used as donor cells. Partial MEFs were subjected to serum deprivation for 18 h and thereafter resupplied with additional serum. Serum-refeeding MEFs (S-MEFs) were then used as iPSC donor cells. We infected MEFs with the four “Yamanaka factors” (pMXs-Oct4, Sox2, Klf4, and c-Myc) and cultured the cells as reported previously for iPSC derivation [15]. Normal adherent fibroblasts were generally elongated and spindle shaped. Serum-starved MEFs spread much less, with moderately decreased cellular size (Fig. 1a, a′). At Day 6, AP^+^ colonies were detected in each group. The primary mature colonies in the serum starvation group were observed at Day 12 (Fig. 1b). In particular, more AP^+^ colonies were obtained in the starvation induction group compared with the normal induction group (*p* < 0.01), suggesting that serum starvation increased the number of AP^+^ colonies (Fig. 1e). After 12–14 days of culture, colonies that resembled mouse ESCs were observed, noted by well-defined phase-bright borders. These colonies were passaged to generate pluripotent cell lines (Fig. 1c, c′).

### Identification of pluripotency

The established S-iPSC lines were then further characterized. P 8 S-iPSCs displayed ESC-like colony morphology, maintaining AP activity (Fig. 1c, c′, d, d′). Pluripotency markers such as Oct4, Nanog, and SSEA1 were expressed (Fig. 1g). Teratomas with all three germ layers determined by H & E staining were observed from S-iPSCs injected into nude mice, indicating a considerable degree of pluripotency (Fig. 1f). These results confirmed that S-iPSCs derived from S-MEFs were reprogrammed into a pluripotent state.

### Q-PCR analysis of rDNA transcriptional activity-related gene expression

**Fig. 2:**
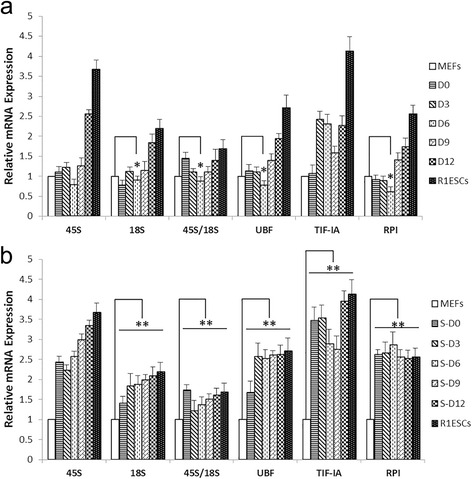
Q-PCR analysis of rDNA-related gene expression during the **a** MEF and **b** S-MEF cell reprogramming process. *Bars* represent relative levels of rDNA-related genes normalized to Gapdh mRNA. Expression level of MEFs was set as 1. Significant differences at *p < 0.05 and **p < 0.01, *n* = 3. *D* day, *ESC* embryonic stem cell, *MEF* mouse embryonic fibroblast, *UBF* upstream binding factor, *TIF-IA* transcription initiation factor IA, *RPI​* RNA polymerase I

Next, we focused on the epigenetic regulation of rDNA transcriptional activity upon reprogramming. Throughout the retroviral infection reprogramming period (12–14 days), Days 0, 3, 6, 9, and 12 were chosen as monitoring days. Cell samples were collected on each certain day, which we believed could stand for the average expression around these days. It is well known that rRNA gene-encoded 45S pre-rRNA can be processed to generate 18S rRNA subsequently [3]. The ratio of 45S/18S would reflect the rDNA transcription condition. Newly synthesized rRNA precursor could be processed to mature rRNAs or be degraded directly [21]. When both the ratio of 45S/18S and 18S rRNA are increased, 45S rRNA synthesis is activated and rDNA transcriptional activity is upregulated. The other way round, decreased 45S/18S and 18S rRNA indicates reduced expression of 45S rRNA, and rDNA transcriptional activity is downregulated [22]. Thus, 45S, 18S, 45S/18S, rDNA transcription factor UBF, transcription initiation factor IA (TIF-IA), and subpopulation of Pol I (RPI) were considered to relate to rDNA transcription activation. At the early stage of iPSC generation, most of the genes expressed in rDNA transcription were downregulated, except 18S and TIF-IA, and were increased steadily up to ESC levels as time went on. Day 6 MEFs had the lowest expression level of 45S/18S, UBF, RPI, and a reduced 18S, indicating the lowest rDNA transcriptional activity during the reprogramming process (Fig. 2a). These data suggested that, after transient inhibition, retroviral-induced rRNA transcriptional activity was reprogrammed towards a pluripotent state during somatic cell reprogramming.

For the serum deprivation group shown in Fig. 2b, regulation of rDNA transcriptional activity-related gene expression was raised significantly against donor cell levels. Expression levels of 45S/18S, 18S, UBF, TIF-IA, and RPI and were upregulated gradually near ESC levels in the late reprogramming process. In view of this different transcriptional outcome, we speculated that serum starvation could facilitate the rDNA transcription. Here, we chose 18 h of starvation in the following experiments according to a previous report [23]. Serum starvation induced cell cycle synchronization, resulting in a notable enrichment of the G0/G1 phase in fibroblasts when compared with normal control (Additional file 2: Figure S1). A total of 76.11 % of fibroblasts were arrested at the G0/G1 phase after starvation. These cell cycle profiles were in agreement with previous studies [23, 24]. Refeeding with 15 % FBS for 14–16 h before infection, a mass of cells was stimulated to enter the cell cycle and started mitosis simultaneously. Therefore, a great demand for rRNA synthesis and rDNA transcription was induced after serum retrieval. These data suggest that cell cycle synchronization stimulated rDNA transcription activation during somatic cell reprogramming.

### Western blot analysis of UBF protein expression

**Fig. 3:**
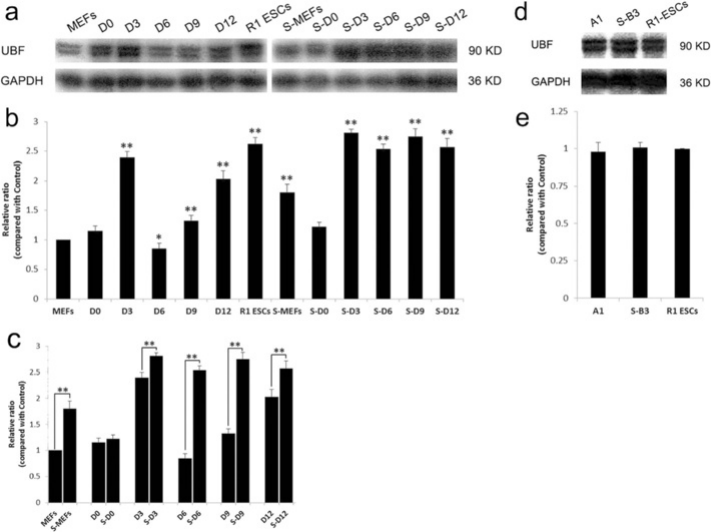
Western blot analysis of UBF proteins during the iPSC and S-iPSC generation process. **a** UBF protein expression during the MEF and S-MEF reprogramming process. **b** Densitometry analysis of UBF proteins during the MEF and S-MEF reprogramming process. Expression level of MEFs was set as 1. **c** Comparison of UBF protein expression between two types of partially reprogrammed cells. **d** UBF protein expression of iPSC line (A1), S-iPSC line (S-B3), and R1 ESCs. **e** Comparison of UBF protein expression between iPSC line (A1), S-iPSC line (S-B3), and R1 ESCs by densitometry analysis. Expression level of R1 ESCs was set as 1. GAPDH was used as an internal reference. The intensities of bands were quantified by densitometry analysis of the Image J analyzer system. Significant differences at *p < 0.05 and **p < 0.01, *n* = 3. *D* day, *ESC* embryonic stem cell, *MEF* mouse embryonic fibroblast, *S-MEF* serum-refeeding MEF, *UBF* upstream binding factor

A previous study has confirmed that UBF expression is reduced in differentiated cells, indicating the regulation of rDNA transcription during growth and differentiation [8]. We also detected the UBF protein expression by western blot throughout the reprogramming procedure (Fig. 3a). The established iPSC lines used here were iPSCs-A1 and S-iPSCs-B3 (Fig. 3d). Obviously, UBF protein expression levels were in accordance with the Q-PCR results already presented (Fig. 2). In the normal induction group, UBF proteins began with a short-lived decline, and were reprogrammed towards the ESC level. Day 6 MEFs had the lowest UBF expression level, suggesting the lowest rDNA transcriptional activity during early reprogramming (Fig. 3b). For the serum starvation group, however, UBF protein expression kept rising during S-iPSC generation (Fig. 3b). We found that the serum deprivation group had significantly higher expression of UBF than that of the control group (Fig. 3c). Furthermore, UBF proteins of iPSCs-A1, S-iPSCs-B3, and R1 ESCs displayed at the same level (Fig. 3e). Eventually, UBF proteins were upregulated to a pluripotent state compromised with the high proliferation demand.

### rDNA methylation detection

**Fig. 4:**
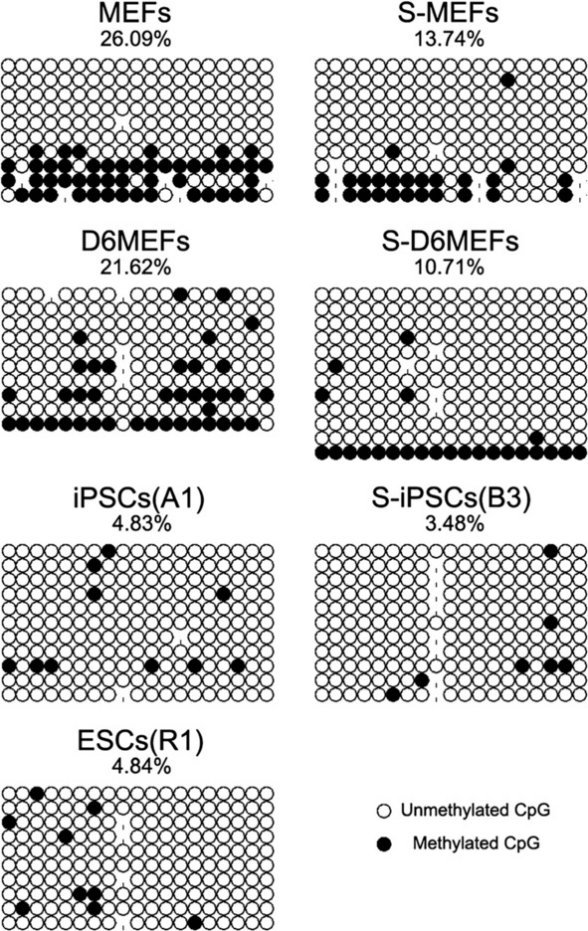
Methylation detection of rDNA promoter regions. rDNA methylation levels of two donor cells (MEFs and S-MEFs), pre-iPSCs (D6 MEFs and S-D6 MEFs), iPSCs (A1), S-iPSCs (B3), and R1 ESCs were evaluated. 5′-ETS (81–527) of the rDNA gene was chosen as the target sequence to evaluate the rDNA methylation levels. The methylated cytosines were counted and compared. *D* day, *ESC* embryonic stem cell, *iPSC* induced pluripotent stem cell, *MEF* mouse embryonic fibroblast, *S-iPSC* S-MEF-generated iPSC, *S-MEF* serum-refeeding MEF

Additionally, rDNA methylation levels of parental cells, pre-iPSCs, and iPSCs were also evaluated. These rDNA methylation results were consistent with previous Q-PCR and western blot results. According to our data, normal MEFs had the highest rDNA methylation level (26.09 %), whereas the synchronized fibroblasts had a lower methylation level at 13.74 %. The evident difference between 26.09 % and 13.74 % was probably due to the active RNA and ribosome synthesis after serum complementary for synchronized cells. Compared with Day 6 S-MEFs (10.71 %), Day 6 MEFs had a higher rDNA methylation level at 21.62 %, which was suitable for the impaired rDNA transcriptional activity (Fig. 4). Meanwhile, our results revealed that iPSCs derived from MEFs and S-MEFs displayed high demethylation in rDNA promoter regions, which was comparable with the high proliferation characteristic of stem cells.

## Discussion

It is widely recognized that complete reprogramming is accompanied by extensive epigenetic remodeling to prevent differentiation and promote self-renewal [15, 16]. Since somatic cell reprogramming requires global epigenetic changes, little is known about the regulation of rDNA transcriptional activity during this process. Here, we detected rDNA transcriptional activity changing during iPSC and S-iPSC generation.

In this study, iPSCs were used to investigate rDNA epigenetic changes occurring in four-factor-mediated reprogramming. We infected MEFs and S-MEFs with “Yamanaka factors” for iPSC derivation. The established S-iPSC lines expressed pluripotency markers, and the differentiation potential was confirmed by teratomas with all three germ layers (Fig. 1). Next, we compared the different rDNA transcription regulation patterns between iPSC and S-iPSC generation. We found that serum deprivation significantly stimulated rDNA transcription level compared with the control group. By the end of reprogramming, both iPSCs and S-iPSCs had acquired upregulated rDNA transcription-related genes (45S/18S, 18S, UBF, TIF-IA, and RPI), UBF proteins, and highly-demethylated rDNA promoter regions, which were corresponding to R1 ESCs (Fig. 2). Cells were distinguished from parental cells with unmodified rDNA transcription profiles, implying active rDNA transcription.

Undoubtedly, donor cells with various differentiation statuses will influence the reprogramming efficiency [25]. Based on our results, the main differences of rDNA epigenetic changes existed within the primary stage of reprogramming: Day 6 MEFs had the lowest expression level of 45S/18S, UBF, RPI, reduced 18S genes, and UBF protein, and a relatively high rDNA methylation level, indicating a low-level rDNA transcriptional activity during early reprogramming. However, the serum starvation group did not undergo a transient rDNA transcriptional inhibition. Conversely, the rDNA transcriptional level was reactivated directly without fluctuation (Fig. 2). We hold the view that serum starvation promoted rDNA transcriptional activity during the early stage of reprogramming. The phenomenon could be partially explained as follows. First, before retrovirus infection, serum-starved MEFs were recovered from 15 % FBS supply. Fibroblasts were released to enter the cell cycle and started mitosis immediately. The activated cell proliferation resulted in a burst of protein synthesis and rDNA transcription within the initial stage of reprogramming. Second, a previous report confirmed that retroviruses required the disassembly of the nuclear envelope at mitosis in order to enter the nucleus and replicate [26]. Chen et al. [23] found that retrovirus-infected synchronized cells prior to the G2/M peak could facilitate retroviral infection efficiency, thereby improving cell proliferation and reprogramming. Above all, this would help explain our results that cell cycle synchronization stimulated rDNA transcription reactivation throughout the reprogramming process.

iPSCs have a striking resemblance to ESCs, including epigenetic marks and the pattern of gene expression [15, 16]. Reprogramming is likely a stochastic process, and epigenetic resetting is essential to overcome the barrier to pluripotency [27]. Cells preserve an intermediate stage with similar morphology to iPSCs and ESCs but without the expression of core pluripotency markers described as partially reprogrammed cells [28–30]. Recent studies have identified these pre-iPSCs by certain classification, such as AP^+^/Oct4-GFP^−^ cells and Thy1^+^/SSEA1^+^ cells [29, 31]. Stable cell sorting and culture of pre-iPSCs contributes to exploring the regulation mechanism that unlocks partially reprogrammed cells into a fully reprogrammed state. Acquisition of a pre-pluripotent state is supposed to occur during the early stage of reprogramming. In our study, we used MEFs and MEFs subjected to serum deprivation as donor cells. A significant increase of AP^+^ colonies was observed at Day 6 in the serum starvation group, compared with Day 6 MEFs (Fig. 1e). During the G2 and S phases, rDNA transcription was actively transcribed, and transcription is maximal during the S and G2 phases [32]. The activated cell proliferation resulted in a burst of protein synthesis and rDNA transcription within the initial stage of reprogramming. Therefore, there was a distinct difference in methylation levels of MEFs and S-MEFs. In the normal induction group, decreased expression levels of 45S/18S, 18S, UBF, RPI, and UBF proteins showed a transient rDNA transcriptional inhibition. On the contrary, expression levels of 45S/18S, 18S, UBF, TIF-IA, RPI, and UBF proteins were remarkably upregulated in the serum pretreated group (Fig. 2b). Most S-MEFs were released to the S phase simultaneously, resulting in a stimulation of rRNA and protein synthesis within the initial stage of reprogramming. After retrovirus infection, rRNA synthesis remained in a persistent active state to support the cells’ unusually accelerated proliferation induced by Yamanaka factor transfection. The rDNA methylation level of Day 6 S-MEFs was 10.71 %, which was dramatically lower than Day 6 MEFs at 21.62 % (Fig. 4). Taken together, Day 6 S-MEFs had relatively high rDNA transcription activation. Increased rDNA transcriptional activity by serum starvation pretreatment may help partially reprogrammed cells overcome the epigenetic barrier, leading to the increase of AP^+^ colonies. The nucleosome remodeling and deacetylation complex (NuRD) is a transcriptional modulator that integrates ATP-dependent chromatin remodeling and histone modifying activities [33]. NuRD has been shown to be required for regulation of Pol I transcription [12]. Recent research considered that the complete erasure of epigenetic mark Mbd3/NuRD was required to modulate ESC transcriptional heterogeneity and maintain ESC lineage commitment [34]. Knockdown of Mbd3/NuRD was sufficient to maintain the pluripotency of ESCs in the absence of LIF and generate fully reprogrammed iPSCs (AP^+^/Oct4-GFP^+^) rather than partially reprogrammed (AP^+^/Oct4-GFP^−^) cells. However, ESCs lacking Mbd3/NuRD would have a restricted differentiation potential [35, 36]. On the contrary, for overexpression of Mbd3/NuRD, cells were trapped in a partially reprogrammed state due to the established heterochromatic features and the silence of ESC-specific marker genes, including Oct4 and Nanog [31]. Methyl-DNA binding domain protein 2 (MBD2) is a member of epigenetic inhibiting factors and could bind to methylated NANOG promoter regions to suppress transcription of NANOG [37]. Overexpressed miR-302 cluster or decreased MBD2 expression was thought to increase NANOG gene expression in cells progressing toward complete reprogramming [38]. Important epigenetic modifications, including diverse histone modifications, are involved in transcription repression and activation [39]. Histone H3 lysine 9 (H3K9) methylation was discovered as an epigenetic determinant for pre-iPSCs to establish and maintain the epigenetic barrier [40]. Above all, proper epigenetic modulation of partially reprogrammed cells might overcome the transition barrier to full reprogramming.

The transcriptional activity of rRNA genes varies between cell types, metabolism conditions, and specific environmental challenges, indicating that epigenetic features change during development and differentiation. Cells under injured metabolism, such as nutrient starvation, oxidative stress, and cell senescence, have an impaired rDNA transcriptional activity, whereas a positive influence that stimulates growth and proliferation upregulates Pol I transcription [13, 41]. In our study, rDNA methylation level of MEFs, S-MEFs, Day 6 MEFs, and Day 6 S-iPSCs were 26.09, 13.74, 21.62, and 10.71 %, respectively (Fig. 4). Firstly, our data showed that normal MEFs had the highest rDNA methylation level (26.09 %). S-MEFs, released from the G0/G1 checkpoint, were involved in active RNA and ribosome synthesis, leading to a lower rDNA methylation level. Secondly, the methylation downtrend between pre-iPSCs and MEFs (26.09 % vs 21.62 % and 13.74 % vs 10.71 %) showed the continuous upregulation of rRNA gene transcription. Lastly, the rDNA methylation level of iPSCs-A1, S-iPSCs-B3, and R1 ESCs were comparable, confirming that iPSCs and S-iPSCs were in accordance with ESCs for high proliferation and active protein synthesis. Somatic cell reprogramming can be accomplished by a variety of ways, such as nuclear transplantation (nuclear transfer) [42, 43], cell fusion [44], and direct reprogramming to pluripotency [16]. Zheng et al. found that MEFs had the highest rDNA methylation level at 22.57 %, ESCs had the lowest (6.76 %), and cumulus cells were in the middle (13.59 %). After nuclear transfer, a nuclear reprogramming strategy, MEFNT embryos preserved the highest methylation level (15.52 %), compared with CCNT embryos (9.67 %) and ESNT embryos (6.36 %), indicating that those methylated rRNA genes in donor cells were not activated fully [19]. Although our methylation data for ESCs and MEFs were not exactly the same as Zheng et al.’s results, we concurred that pluripotent stem cells had more cordial rDNA transcription activity than MEFs, and cell reprogramming would recover rDNA epigenetic statuses of donor cells to varying degrees. However, there were opposite opinions over rDNA epigenetic remodeling involved in adult cell reprogramming. Xenopus egg extract-mediated nuclear reprogramming has been shown to induce remodeling of chromatin and reprogram gene expression in somatic cells [45, 46]. A previous study showed that egg extract elicited remodeling of the nuclear envelope, chromatin, and nucleolus, and resulted in a rapid and stable decrease of ribosomal gene transcription. The downregulation of rDNA transcriptional activity here was distinct from a stress response [47]. Ling et al. [48] believed that cell programming in fact negatively influenced rRNA synthesis and methylation at rDNA promoters was increased in iPSCs as well as in mESCs compared with MEFs. Taken together, it is profoundly suggested that the distinct rDNA transcriptional phenomena hidden behind these diverse reprogramming process require further investigation. The complicated rDNA epigenetic regulatory mechanisms may not be simplified and idealized as a simplified model.

## Conclusion

We demonstrated that cell cycle synchronization could stimulate rDNA transcription reactivation during somatic cell reprogramming into iPSCs. Our findings offer new insights into the regulation of rDNA transcriptional activity during somatic cell reprogramming and will benefit partially reprogrammed cells to overcome the epigenetic barrier to pluripotency.

## Abbreviations

AP, alkaline phosphatase; CCNT, cumulus cell nuclear transfer; DAPI, 4′,6-diamidino-2-phenylindole; ESC, embryonic stem cell; ESNT, embryonic stem cell nuclear transfer; H & E, hematoxylin and eosin; H3K9, histone H3 lysine 9; iPSC, induced pluripotent stem cell; MBD2, methyl-DNA binding domain protein 2; MEFNT, mouse embryonic fibroblast nuclear transfer; MEF, mouse embryonic fibroblast; NuRD, nucleosome remodeling and deacetylation complex; Pol I, RNA polymerase I; Q-PCR, quantitative PCR; rRNA, ribosomal RNA; S-iPSC, S-MEF-generated iPSC; S-MEF, serum-refeeding MEF; TIF-1A, transcription initiation factor 1A; UBF, upstream binding factor

## Additional files

Additional file 1: Table S1. Presenting primers used in rDNA-related gene expression detection of cells. (DOCX 20 kb) Additional file 2: Figure S1. Showing MEF and S-MEF cell cycle distribution analysis. (DOCX 152 kb)
