# Supplementary material for: Serum starvation-induced cell cycle synchronization stimulated mouse rDNA transcription reactivation during somatic cell reprogramming into iPSCs
Source: Stem Cell Res Ther. 2016 Aug 11;7:112. doi: 10.1186/s13287-016-0369-1 (PMC4981958; doi:10.1186/s13287-016-0369-1)
Supplement: Additional file 1: Table S1. — Presenting primers used in rDNA-related gene expression detection of cells. (DOCX 20 kb) [file 13287_2016_369_MOESM1_ESM.docx]

**Additional file 1: Table S1**

**Table S1.** Primers used in rDNA related genes expression detection of cells

| Gene | Gene Identity | Primer sequences (5’~3’) | | E | R^2^ |
| --- | --- | --- | --- | --- | --- |
| 45S rRNA | ID: 100861531 | Sense | CTCCTGTCTGTGGTGTCCAA | 93.0 | 1.000 |
|  | AL592188 | Antisense | GCTGGCAGAACGAGAAGAAC |  |  |
| 18S rRNA | ID: 19791 | Sense | CGCGGTTCTATTTTGTTGGT | 101.3 | 0.999 |
|  | BK000964 | Antisense | AGTCGGCATCGTTTATGGTC |  |  |
| UBF | ID: 21429 | Sense | GGAGGAGGAAGAGGAAGAT | 99.5 | 0.997 |
|  | NM_001044383.2 | Antisense | CGTCGTCGTCATCATCAT |  |  |
| Tif-1A | ID: 106298 | Sense | CAGAGGTCTCGACCCTGAAC | 95.4 | 0.997 |
|  | NM_001039521.1 | Antisense | GTCCGAGTCCTTCCACTTCA |  |  |
| RPI | ID: 19895 | Sense | GAGCTCTGAGCACTGGAGAGA | 98.2 | 0.999 |
|  | NM_009075.2 | Antisense | CCACCCTGGCACATGAAT |  |  |
| Gapdh | ID: 14433 | Sense | GCTTGCTGGTGAAAAGGACCTCTCGAAG | 88.9 | 0.999 |
|  | NM_013556.2 | Antisense | CCCTGAAGTACTCATTATAGTCAAGGGCAT |  |  |

.
