# Supplementary material for: Serum starvation-induced cell cycle synchronization stimulated mouse rDNA transcription reactivation during somatic cell reprogramming into iPSCs
Source: Stem Cell Res Ther. 2016 Aug 11;7:112. doi: 10.1186/s13287-016-0369-1 (PMC4981958; doi:10.1186/s13287-016-0369-1)
Supplement: Additional file 2: Figure S1. — Showing MEF and S-MEF cell cycle distribution analysis. (DOCX 152 kb) [file 13287_2016_369_MOESM2_ESM.docx]

**Additional file 2: Figure S1**

**Figure S1.** MEFs and S-MEFs cell cycle distribution analysis. a Normal MEFs cell cycle distribution analysis. b A summary of cells in each phase of cell cycle counted by relative percentage. c MEFs cell cycle distribution analysis after serum starvation for 18 h. d A summary of cell cycle distribution after serum starvation for 18 h. Compared with MEFs, the data showed cell cycle arrested at G0/G1 phase after serum deprivation for 18 h.
